# Chromosomal aberrations and early mortality in a non-mammalian vertebrate: example from pressure-induced triploid Atlantic salmon

**DOI:** 10.1038/s41437-024-00727-9

**Published:** 2024-10-05

**Authors:** Aurélien Delaval, Kevin A. Glover, Monica F. Solberg, Per Gunnar Fjelldal, Tom Hansen, Alison C. Harvey

**Affiliations:** 1 Institute of Marine Research, Bergen, Norway; 2 Institute of Marine Research, Matre Research Station, Matredal, Norway

**Keywords:** Animal breeding, Development, PCR-based techniques, Ecological genetics

## Abstract

In commercial aquaculture, the production of triploid fish is currently the most practical approach to prevent maturation and farm-to-wild introgression following escapes. However, triploids often exhibit poor welfare, and the underlying mechanisms remain unclear. Inheritance issues associated with sub-optimal hydrostatic pressure treatments used to induce triploidy, or the genetic background of parental fish, have been speculated to contribute. We tested this by quantifying the frequency and type of chromosomal aberrations in Atlantic salmon subjected to a gradient of sub-optimal pressure treatments (Experiment 1) and from multiple mothers (Experiment 2). From these experiments, we genotyped a subsample of ~900 eyed eggs and all ~3300 surviving parr across ~20 microsatellites. In contrast to the low frequency of chromosomal aberrations in the diploid (no hydrostatic pressure) and triploid (full 9500 PSI treatment) controls, eyed eggs subjected to sub-optimal pressure treatments (6500–8500 PSI) had a higher incidence of chromosomal aberrations such as aneuploidy and uniparental disomy, corresponding to lower triploidization success and higher egg mortality rates. We also observed maternal effects on triploidization success and incidence of chromosomal aberrations, with certain half-sibling families exhibiting more aberrations than others. Chromosomal aberrations were rare among surviving parr, suggesting a purge of maladapted individuals during early development. This study demonstrates that sub-optimal hydrostatic pressure treatments and maternal effects not only influence the success of triploidization treatments, but may also affect the incidence of chromosomal aberrations and early mortality. The results have important implications for aquaculture breeding programs and their efforts to prevent farm-to-wild introgression.

## Introduction

Chromosomal aberrations, abnormalities in the structure or number of chromosomes, are the primary cause of spontaneous abortions and a variety of genetic disorders in humans and potentially other species. In most diploids, organisms with two sets of chromosomes, one chromosome set is normally inherited from each parent. However, deviations from the precise cellular processes involved in cell division during meiosis or mitosis can lead to the unequal segregation of chromosomes (nondisjunction). The result is a chromosome number that deviates from an exact multiple of the haploid set, known as aneuploidy (Orr et al. 2015; Taylor et al. 2014), which can lead to cellular imbalances that reduce cellular and organismal fitness (Williams et al. 2008). In humans, aneuploidy occurs in at least 5% of all pregnancies and is the primary cause of spontaneous abortions (Hassold and Hunt 2001; Torres 2023). Only a few forms of aneuploidy are compatible with human life, however, these usually have clinical consequences in the form of genetic syndromes (e.g., trisomy 21 or Down syndrome), mosaic variegated aneuploidy, sterility, and possibly tumorigenesis (Orr et al. 2015; Torres 2023; Williams et al. 2008).

The association between chromosomal aberrations and spontaneous abortions and pathologies have also been observed in domesticated mammals like equines (Shilton et al. 2020), bovines (Schmutz et al. 1996), and in mouse models (Williams et al. 2008). Studies on humans (Taylor et al. 2014; Thomas et al. 2021; Webster and Schuh 2017), other mammals (Rizzo et al. 2019), and even the model nematode *Caenorhabditis elegans* (Toraason et al. 2022) have demonstrated that variables relating to the maternal germline, and in particular oocyte age, may increase the probability of nondisjunction during meiosis. The current evidence, while mostly limited to humans and domesticated mammals, suggests that chromosomal aberrations and their impacts on fitness may be prevalent across taxa, and may be predisposed by genetic and environmental factors.

In fish and shellfish aquaculture, artificial ploidy manipulation is frequently used to improve production efficiency and to address environmental issues (Piferrer et al. 2009). The most common approach is the use of a hydrostatic pressure shock at precise timings following fertilization, a technique that has been used to produce triploids, tetraploids, and homozygotic clonal lines (Chourrout 1984; Hansen et al. 2020; Piferrer et al. 2009). In the production of sterile triploids, the treatment normally causes the retention of the second polar body during meiosis II and results in offspring with two sets of chromosomes inherited from the dam and one from the sire. This approach is primarily intended as a biocontainment strategy to prevent farm-to-wild introgression following escape events (Benfey 2016), which is currently considered a major environmental sustainability issue for the Atlantic salmon (*Salmo salar*) farming industry (Glover et al. 2017). The re-allocation of energy from gonadal development to somatic growth also offers production advantages when using triploids.

Despite the appeal of triploid Atlantic salmon production to minimize introgression and potentially enhance farm productivity, triploids occasionally demonstrate poorer growth, welfare, and higher mortality rates than diploids when reared in suboptimal conditions (Fraser et al. 2012; Madaro et al. 2022; Stien et al. 2019), acting as a disincentive to their use. Although solutions have been introduced to overcome some of these challenges, such as the addition of nutritional supplements to fish feed and adjusting water temperature to prevent cataracts and bone deformities (Fjelldal et al. 2016; Fraser et al. 2015; Sambraus et al. 2017), triploid Atlantic salmon still occasionally demonstrate compromised welfare, and the mechanisms behind this are still not fully understood. The fish welfare implications are such that the use of triploids in the Norwegian aquaculture industry is currently under evaluation (VKM 2023), however triploids are already used in commercial production in Australia (Dempster et al. 2016) and Canada (DFO 2016). Alternative methods to produce sterile fish are being developed, including gene modification (Güralp et al. 2020; Kleppe et al. 2020; Wargelius et al. 2016) and gene regulation (Andersen et al. 2022) technology, however the hydrostatic pressure treatment is currently the most affordable, scalable, and publicly accepted approach.

Under controlled experimental conditions, the success rate of hydrostatic pressure treatments in producing triploids is generally considered high (>95%, Glover et al. 2020; Jacq 2021). However, at larger commercial scales, triploidization success is more variable and has been reportedly as low as 44% (Stien et al. 2019). Some experiments have documented the occurrence of aneuploids and mosaic individuals following the pressure shock treatment (Fujimoto et al. 2013; Káldy et al. 2021; Yamazaki and Goodier 1993; Yang et al. 2000). Furthermore, studies on coho salmon *Oncorhynchus kisutch* (Devlin et al. 2010) and Atlantic salmon (Glover et al. 2020) have revealed that pressure-induced triploids can sometimes display chromosomal aberrations, with incomplete retention of paternal or maternal chromosomes, even when triploidization success is apparently high. We therefore hypothesized that, in instances where the hydrostatic pressure treatment is not optimally applied to all eggs in large commercial batches, the treatment may not only cause a reduction in triploidization success but also inadvertently lead to these occasional inheritance anomalies. Because earlier work has also suggested a possible effect of variables relating to the parental strain and egg quality on the success of triploidization treatments and of resulting phenotypes (Galbreath and Samples 2000; Harvey et al. 2017), we also hypothesized that there may be a maternal effect on the incidence of chromosomal aberrations following pressure-shock treatments. An association between the pressure shock treatment, parental origin or strain, and chromosomal aberrations may have important practical implications for breeding programs that use this technique to manipulate ploidy, particularly when sterility and good animal welfare are the desired outcomes. Therefore, an understanding of the consequences of this approach and any improvements that can be made in its application warrant further investigation, as it still represents an important tool in the aquaculture industry.

To investigate this, we recently developed a microsatellite analysis technique that considers allele dosage information to estimate ploidy and inheritance patterns in diploid and triploid Atlantic salmon (microsatellite DNA allele counting—peak ratios, or MAC-PR, Delaval et al. 2024). The results identified aneuploid eyed eggs within experimental batches produced using standard diploid and triploid treatments, which would have gone undetected if using common (qualitative) microsatellite and cytological ploidy verification tools. In the present study, we applied this technique to test whether suboptimal hydrostatic pressure treatments and the maternal germline affect the occurrence of chromosomal aberrations. We performed two experiments in which Atlantic salmon eggs were subjected to a gradient of suboptimal pressure treatments, using a half-sibling experimental design (multiple dams crossed with one sire). We evaluated the ploidy and inheritance patterns among eyed eggs and surviving parr to assess any associations with early mortality.

## Materials and methods

### Family production and experimental design

Two complimentary experiments were initiated on 18 November 2021 at the Matre Research Station, Institute of Marine Research (IMR), Norway. Twelve domesticated dams from the commercial Mowi strain (from the Mowi breeding station in Askøy, Norway) were simultaneously crossed with one sire of admixed origin (75% wild, 25% farmed, also from the Mowi strain) to create 12 half-sibling families. Offspring from three of these families (families 1–3) were used in Experiment 1, and offspring from all 12 families were used in Experiment 2.

Experiment 1 consisted of five treatments, each performed in two replicates: a negative diploid control (no pressure treatment, 0 PSI), three intermediate pressure treatments (6500 PSI, 7500 PSI, and 8500 PSI), and a positive triploid control (standard 9500 PSI treatment). The hydrostatic pressure treatments were performed at 37 min 30 s post-fertilization for a duration of 6 min 15 s, following standard procedures for triploidy induction (Glover et al. 2020; Harvey et al. 2017). Initially, approximately 200–300 eggs per female were used per treatment replicate and incubated in separate family treatment replicates. In week 3 of 2022, a sub-sample of 15 eyed eggs per family in each treatment replicate (totaling 450 eggs) was collected and stored in 100% ethanol for genotyping to verify ploidy and chromosomal aberrations. A further 100 eggs per family from each treatment replicate were picked to be reared until the parr stage, totaling 3000 individuals, separated across 10 tanks by treatment replicate.

Experiment 2 consisted of two treatments: 0 PSI (standard diploid) and 9500 PSI (standard triploid, produced under the same conditions as in Experiment 1). Initially, approximately 350 eggs per female were used per treatment and incubated in separate family units (24 units in total). In week 3 of 2022, 100 eggs per family unit were picked and mixed into 2 replicate tanks per treatment (i.e., 600 juveniles per tank from a mix of families, totaling 2400 juveniles). A sub-sample of 20 eggs per family-treatment unit (totaling 480 eggs) was collected as above for genotyping.

For both experiments, eggs were kept at 6 °C in the hatchery from fertilization until start feeding in week 16. The fish were produced as underyearling (out-of-season) smolts using a “square wave” photoperiod to induce off-season smoltification (Thrush et al. 1994), with 6 weeks of light-day (LD) 12:12 at ambient water temperature from week 33 to 39 of 2022 to simulate winter followed by 350 degree-days with LD24:0 to simulate summer. In week 36 of 2022 (3 weeks into the LD12:12 regime), a sample of 1106 parr (pre-smolts) from Experiment 1 and 2196 parr (pre-smolts) from Experiment 2 had their adipose fins clipped and stored in 100% ethanol for genotyping. Mortality data was recorded from fertilization until egg picking, from egg picking until start-feeding, and then until sampling at the parr stage in week 36. All fish from Experiments 1 and 2 continued to be reared until harvest size as part of an ongoing experiment to assess their growth and welfare.

### Genotyping

DNA isolation and microsatellite genotyping were performed at IMR’s molecular genetic laboratory in Bergen, Norway. Genomic DNA was isolated on a Biomek i5 Automated Workstation using the DNAdvance isolation kit (Beckman Coulter), DNA concentration measured with a NanoDrop 1000 Spectrophotometer (Thermo Fisher Scientific), and DNA concentrations standardized to approximately 16–20 ng/µL using a Freedom EVOware robot (Tecan) prior to PCR. All samples, including the 13 parents, were genotyped at 30 microsatellite loci (*SSsp2210, SSspG7, SsaD144, Ssa202, Sp2201, SsaD157, Ssa289, Ssa14, Sp1605, Ssa171, Sp2216, SsaF43, Ssa197, SSsp3016, MHC1, MHC2, SsOSL85, Ssa412, Ssa405, Ssa98, SsOSL25, SSsp2215, EST107, EST68, EST28, EST19, Ssa407, Ssleer15.1, Sleen82*, and *Sleer53*). These were divided across five multiplex PCR reactions. Further details about these markers and PCR protocols are provided in Supplementary Tables S1–S3. PCR products were analyzed on an ABI 3730 Sequencer (Applied Biosystems) and sized using a 500LIZ^TM^ size standard. This microsatellite panel has been used extensively on Atlantic salmon in this laboratory (Harvey et al. 2017, 2019; Madhun et al. 2023; Ozerov et al. 2017).

Alleles were automatically binned and manually checked by two persons in GeneMapper Software 6. A recently developed bioinformatic application of the Microsatellite DNA Allele Counting–Peak Ratios technique (MAC-PR, Delaval et al. 2024), which builds on the principles described by Esselink et al. (2004), was used to infer the somy status (disomic or trisomic) and allelic configuration (AB, AAB, ABB, or ABC) of each heterozygous marker for every individual. The MAC-PR method considers the relative signal intensity (peak height) of alleles on electropherograms to infer the copy number (or dosage) of each allele. Because not all markers will be informative due to the occurrence of background noise such as artifact peaks and stutter, the method involves a careful screening protocol to identify informative loci. As such, the ploidy of every individual was determined by the proportion of informative heterozygotic markers that were clearly trisomic (genotypes AAB, ABB or ABC) rather than disomic (genotype AB). Individuals were considered diploid if this proportion was 0, triploid if this proportion was 1, and “aneuploid” if this proportion was between 0 and 1 (i.e., the individual had a mix of disomic and trisomic markers). All samples identified as “aneuploids” were checked in GeneMapper for a third time to rule out genotyping error. From the resulting genotypes, individuals were excluded if they had low genotyping success (<50% scored markers) prior to data analysis. Homozygous markers, for which dosage cannot be determined with this technology, were not informative for ploidy assessment, but were informative for family assignment and to check for chromosomal aberrations.

The genotypes were used to assign the parr from both experiments to family using a modified version of Family Analysis Program (FAP, Delaval et al. 2024). FAP is an Excel-based program originally developed by Taggart (2007) that uses an exclusion-based algorithm to assign offspring to their families of origin, and can accommodate both diploid and triploid families. Family assignment of the eggs was already known, as families were separated in hatchery units. Next, we applied an in-house script, also described in Delaval et al. (2024), to identify inheritance aberrations. The script identifies any genotypic mismatches between offspring and their parents, and reports on the likely nature of the error. The program can identify the following types of errors: (1) missing paternal and/or maternal allele, (2) a trisomic genotype where two alleles are paternally inherited, which is inconsistent with polar body retention that normally results from the hydrostatic pressure treatment, and (3) a base pair shift caused by slippage of the microsatellite repeat motif. All inheritance errors identified by the program were checked in GeneMapper for a third time to exclude genotyping error.

### Statistical analysis

Statistical analyses were performed in R (v. 4.2.2, R Core Team 2022). We tested for the effects of the hydrostatic pressure treatment, family origin, and developmental stage (eyed egg or parr) on the ploidy status of the offspring. Two statistical tests were performed for each dataset, one with the response variable “triploid” (1 for triploid, 0 for non-triploid) and one with the response variable “aneuploid” (1 for aneuploid, 0 for non-aneuploid). Both response variables were Bernoulli distributed but modeled using the binomial family. Because the samples in Experiment 1 were nested across ten treatment replicates, we implemented generalized linear mixed-effects models (GLMMs, modeled using the package glmmTMB, Brooks et al. 2017) on this dataset, with pressure treatment, family and developmental stage as fixed effects, and treatment replicate as a random effect. Generalized linear models (GLMs) were used for Experiment 2, with pressure treatment, family, and developmental stage as fixed effects; this dataset was not nested, as there was only one replicate per treatment.

The significance of the fixed effects was evaluated using the *drop1* function based on AIC values (Bolker et al. 2009). When two models performed similarly, the most parsimonious model was retained while always retaining the random nesting variable. Model fit was evaluated by plotting the model residuals against the fitted values and the model covariates using the package DHARMa (Hartig 2022).

## Results

### Experiment 1: ploidy

Twenty loci passed the quality-checking requirements for MAC-PR and were deemed informative for ploidy assessment in Experiment 1 (*SSsp2210, Sp2201, SsaD157, Ssa14, Sp1605, Sp2216, SsaF43, Ssa197, SsOSL85, Ssa412, SsOSL25, SSsp2215, EST107, EST68, EST28, EST19, Ssa407, Ssleer15.1, Sleen82* and *Sleer53*). After excluding individuals with low (<50%) genotyping success across these 20 markers, the resulting dataset comprised of 438 egg genotypes and 1098 parr genotypes.

**Fig. 1:**
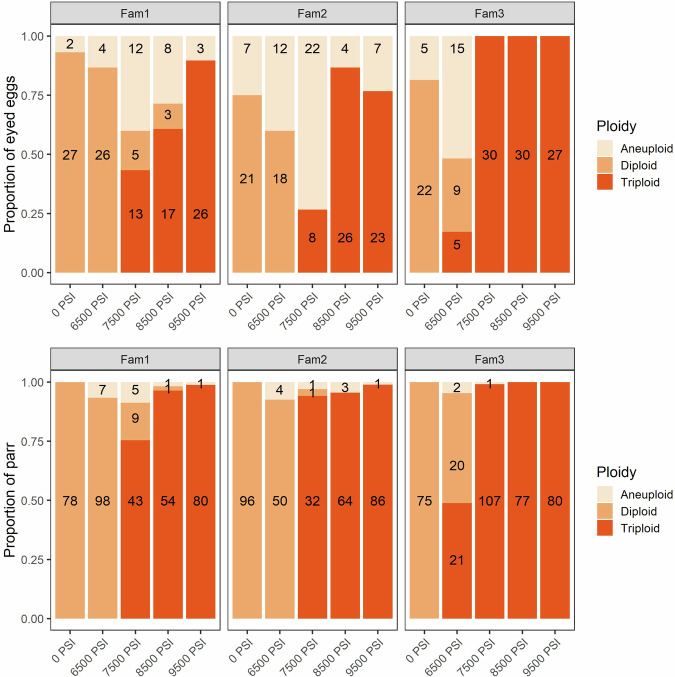
The effect of sub-optimal hydrostatic pressure treatments on Atlantic salmon ploidy (Experiment 1). The proportion of diploid, triploid, and aneuploid offspring from three half-sibling families following five hydrostatic pressure treatments. Top: the proportion of eyed eggs (*N* = 437). Bottom: the proportion of surviving parr (*N* = 1098). The number of offspring in each category is shown.

Statistical analyses indicated a significant effect of all three predictors (pressure treatment, family origin, and developmental stage) on the incidence of triploids and aneuploids (Table S4). In Experiment 1, quasi-complete separation led to model convergence issues when triploid was used as a response variable, generating large standard errors. This could be explained by the total absence and/or 100% occurrence of triploids in certain treatment-family combinations (e.g., no triploids were detected in the 0 PSI treatment, Fig. 1). We therefore repeated this statistical model after dropping the 0 PSI treatment (Table S4).

Triploid induction success decreased with decreasing hydrostatic pressure, a pattern that was reflected in both the eyed egg and parr genotypes (Fig. 1 and Supplementary Fig. 1). Triploidization success in eggs was >~80% at 8500 PSI and above, decreasing to 57% at 7500 PSI, and to 6% at 6500 PSI. The incidence of triploids was higher among the parr, even at intermediate pressure treatments; >97% of fish were triploid at 8500 PSI and above, dropping to 91% in the 7500 PSI group and 10% in the 6500 PSI group. The incidence of triploids differed among families, with nearly 100% triploidization success in family 3 at and above 7500 PSI (apart from one aneuploid parr at 7500 PSI). One egg from Family 1, subjected to 8500 PSI, was homozygous at all loci, so ploidy could not be determined for this individual.

Aneuploid eggs occurred at higher frequencies in the 6500 PSI (35%) and 7500 PSI (38%) treatments compared to the other three treatments (12–17%) and were most prevalent in Families 1 and 2 (Fig. 1). The frequency of aneuploids reduced between the egg and the parr stage: no aneuploids remained in the 0 PSI group, and the frequency of aneuploids decreased to frequencies ranging from <1% in the 9500 PSI group to 6% in the 6500 PSI group. Family 3 had the fewest aneuploids. On looking at the degree of aneuploidy (ratio of disomic and trisomic loci) among the 101 aneuploid eggs, 42 (42%) were either trisomic at only 1 locus (they were otherwise diploid) or disomic at only 1 locus (they were otherwise triploid). Among the 26 aneuploid parr, this proportion increased to 69% (18 individuals). This pattern indicates a reduction in the proportion of aneuploids with more intermediate, or severe, forms of aneuploidy (e.g., 25–75% of loci trisomic). The more intermediate degrees of aneuploidy were most prevalent in eggs subjected to sub-optimal hydrostatic pressures, in particular the 6500 PSI and 7500 PSI treatments, and least prevalent in the 0 PSI diploid control group (Supplementary Fig. 2).

### Experiment 2: ploidy

For Experiment 2, 19 markers passed the quality-checking requirements for MAC-PR and were retained in the analysis. These were the same markers as for Experiment 1, except for *Sp2201*; the added genetic diversity from the 12 families meant that certain allelic configurations could not be resolved reliably for this marker, so it was excluded from the analysis. After excluding individuals with low (<50%) genotyping success across these 19 markers, the resulting dataset comprised of 472 egg genotypes and 2179 parr genotypes.

**Fig. 2:**
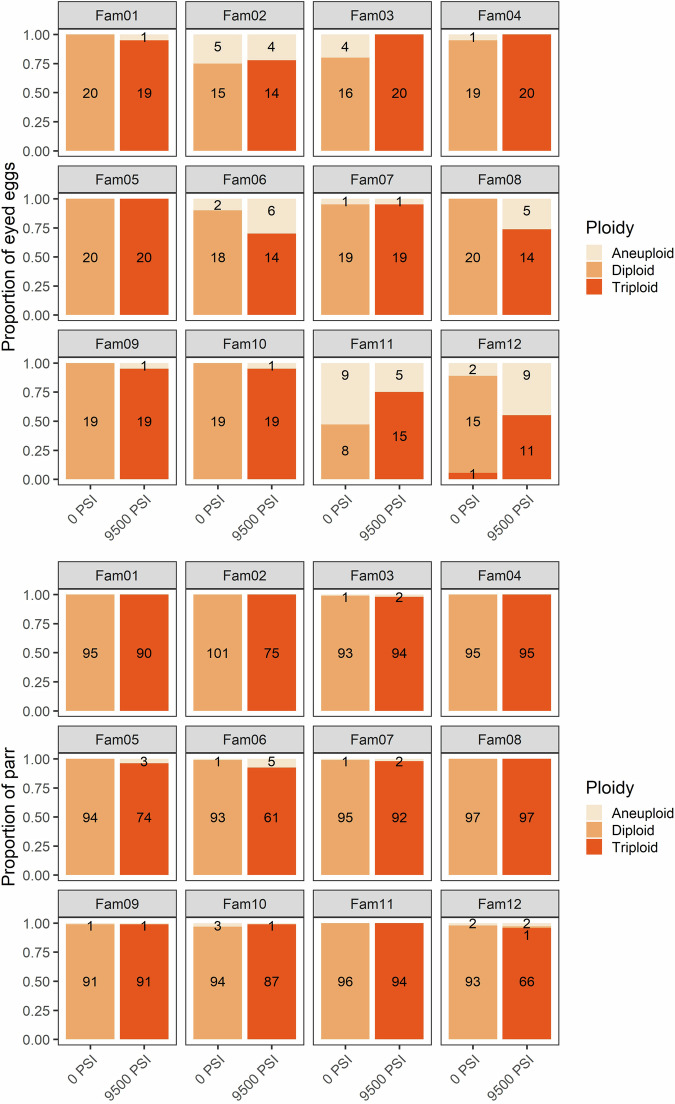
The family effect on Atlantic salmon ploidy following standard diploid and triploid treatments (Experiment 2). The proportion of diploid, triploid, and aneuploid offspring from 12 half-sibling families following standard diploid (no hydrostatic pressure) and triploid (full hydrostatic pressure) treatments. Top: the proportion of eyed eggs (*N* = 470). Bottom: the proportion of surviving parr (*N* = 2179). The number of offspring in each category is shown.

As in Experiment 1, the results of the GLM also indicated a significant effect of all three predictors (pressure treatment, family origin, and developmental stage) on the incidence of triploids and aneuploids (Table S4). Triploidization success was 86% among eyed eggs subjected to 9500 PSI, increasing to 98% in the surviving parr. Only one triploid egg was detected among all 1379 eggs and parr in the 0 PSI treatment. Aneuploidy occurred in 10% of eggs from the 0 PSI treatment, and 14% of eggs from the 9500 PSI treatment (Supplementary Fig. 3). These were spread across 11 of the families, with a higher prevalence in families 2, 6, 11, and 12 (Fig. 2). Only 25 aneuploids were detected among the 2179 parr, representing <1% and <2% of parr from the 0 PSI and 9500 PSI groups, respectively, and spread across seven of the 12 families. Two eggs from the 9500 PSI treatment, both from Family 2, were homozygous across all loci and so their ploidy could not be determined.

### Chromosomal aberrations

**Fig. 3:**
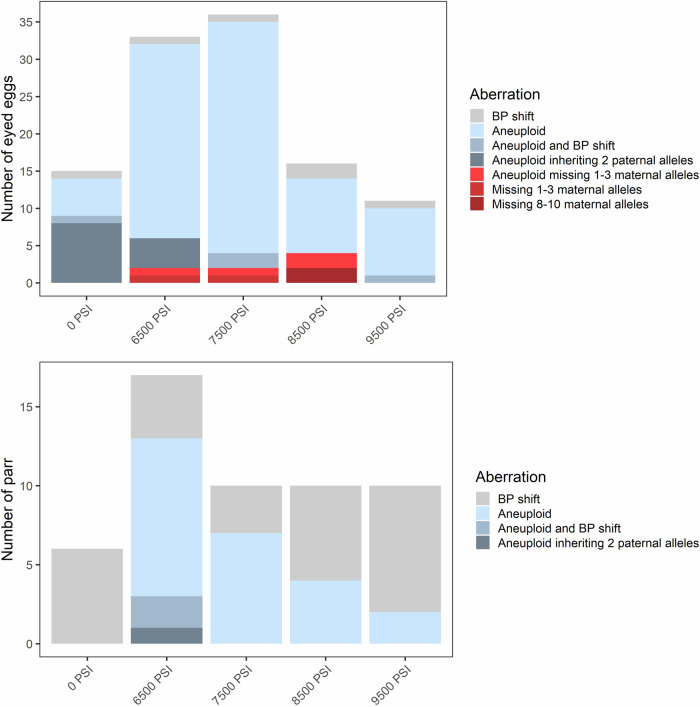
Inheritance aberrations following five hydrostatic pressure treatments (Experiment 1). The frequency and types of inheritance aberrations are shown for eyed eggs (top, *N* = 438) and surviving parr (bottom, *N* = 1098) across all three (pooled) half-sibling families.

**Fig. 4:**
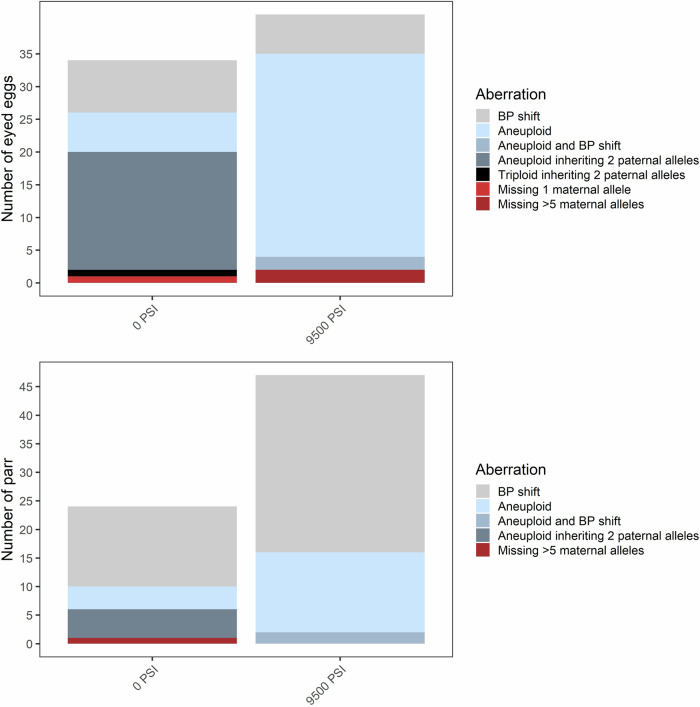
Inheritance aberrations following standard diploid and triploid hydrostatic pressure treatments (Experiment 2). The frequency and types of inheritance aberrations are shown for eyed eggs (top, *N* = 472) and surviving parr (bottom, *N* = 2179) from across all 12 (pooled) half-sibling families.

In addition to aneuploidy, we evaluated offspring genotypes against their parents’ and categorized chromosomal aberrations into four types: (1) offspring lacking the maternal allele at one or a few loci, (2) offspring lacking the maternal allele across multiple (>3) loci, (3) offspring inheriting 2 paternal alleles at a locus, which is inconsistent with polar body retention, and (4) a base-pair shift mutation likely due to slippage of the repeat motif. In some instances, individuals displayed a combination of these chromosomal aberration types (e.g., a base-pair shift at a locus in addition to aneuploidy), and these are summarized in Figs. 3 and 4. Base-pair shifts of one or two repeat motifs occurred among ~2–3% of eggs and parr (Figs. 3 and 4) and across all families (Supplementary Figs. 4 and 5). Because base-pair shifts are a common and well documented occurrence in microsatellites (Goldstein and Pollock 1997; Li et al. 2002) that are unlikely affected by the treatment itself (Glover et al. 2020), we do not consider this error further.

Another frequent error was the occurrence of trisomic loci where two alleles were inherited from the father. This error occurred only in the 0 PSI and 6500 PSI treatments and across all three families in Experiment 1 (Fig. 3 and Supplementary Fig. 4), and only in the 0 PSI treatment and across six of the families in Experiment 2 (Fig. 4 and Supplementary Fig. 5). The error occurred almost exclusively in aneuploid individuals with spontaneous trisomy at 1–2 loci where the individual was otherwise diploid. Across both experiments, this occurred in 34 out of the 80 individuals which displayed 1–2 trisomic loci; the other 46 likely inherited two alleles from the mother. However, there were three noteworthy exceptions: inheritance of two paternal alleles occurred in two eggs from the 6500 PSI treatment in Experiment 1, where trisomy occurred at 3 and 5 loci (both inheriting 2 paternal alleles at one locus), and in the only triploid egg from the 0 PSI treatment in Experiment 2 (with 2 paternal alleles inherited at two loci). The occurrence of this aberration decreased substantially between the egg stage (~3–4%) and the parr stage (<0.3%) in both experiments, consistent with the loss of aneuploids.

In Experiment 1, offspring lacking maternal alleles occurred in eight eggs (~2%), all of which were from the intermediate pressure treatments (6500 PSI to 8500 PSI) and across all three families (Fig. 3 and Supplementary Fig. 4). In two cases, both from Family 1 and at 8500 PSI, the egg lacked the maternal allele across >8 loci. One of these individuals was homozygous across all 20 loci and was flagged for the absence of the maternal allele at 10 loci; at the remaining 10 loci, both parents shared an allele, so no error could be detected. This indicates a total lack of maternal DNA in this individual. Similarly, the second individual, which lacked maternal DNA at 8 loci, was homozygous at 19/20 markers; the one heterozygous marker displayed normal inheritance from both parents. Individuals lacking maternal DNA were not detected among any of the surviving parr.

In Experiment 2, offspring lacking maternal alleles were identified in three eggs (<1%), two of which were from the 9500 PSI treatment and were lacking maternal DNA across multiple loci (Fig. 4). These two eggs were also homozygous across all loci, indicating a total lack of maternal DNA. One surviving parr from the 0 PSI treatment also lacked maternal DNA across >5 loci. Homozygosity was high in this individual (12/19 loci), and at heterozygous loci not flagged by the program, the parr had alleles that were shared by both parents. It is therefore possible that this individual lacked maternal DNA at all markers. In Experiment 2, the more substantial aberrations (lacking multiple maternal alleles) occurred only in Family 2 (Supplementary Fig. 5). However, the assignment of the parr in question to family is questionable, given the presumed absence of maternal DNA in this individual. As in Experiment 1, there was a reduction in aberrations observed between the eyed egg and the surviving parr.

### Mortality

**Fig. 5:**
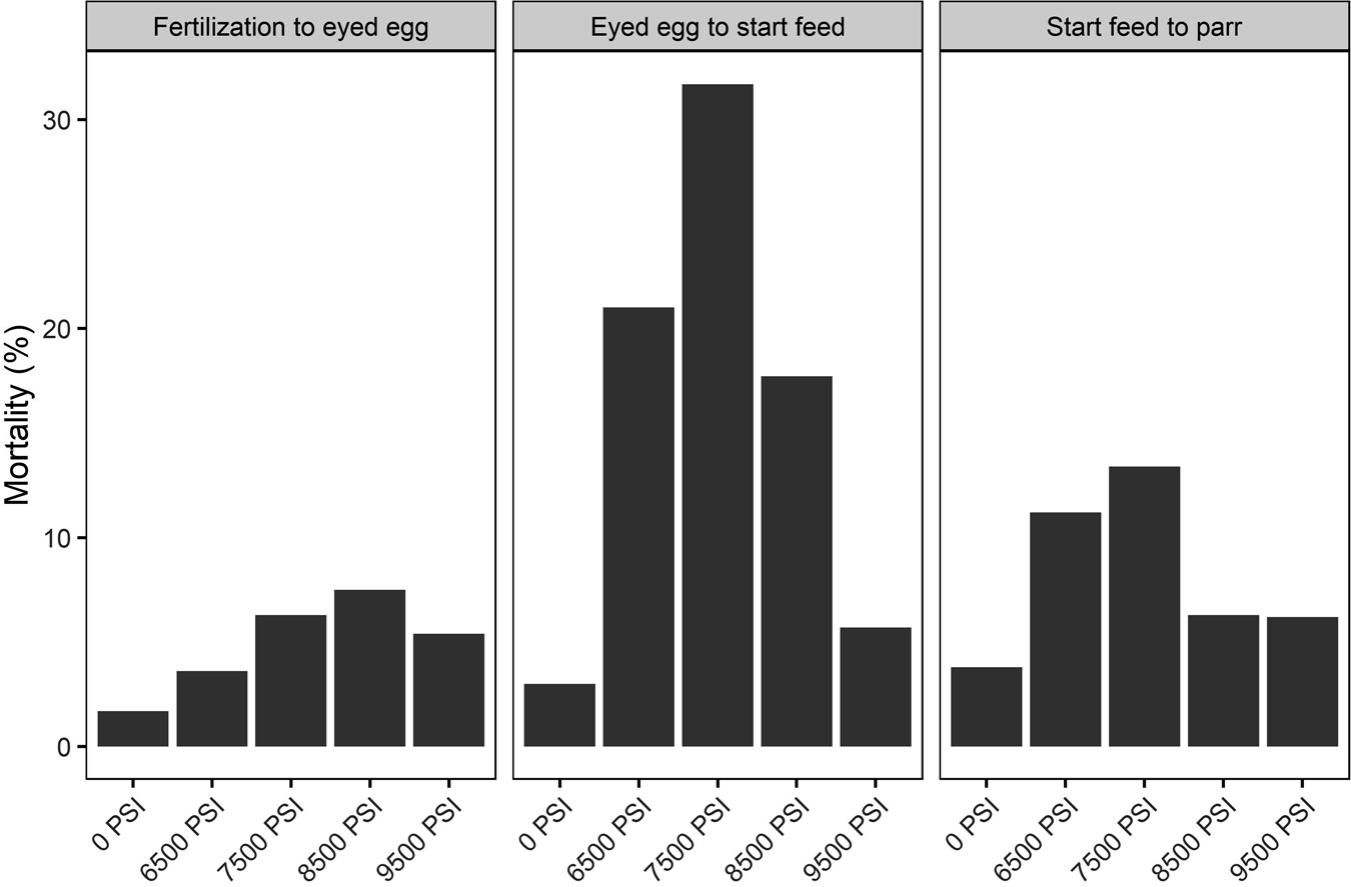
Mortality rates during early life stages following five hydrostatic pressure treatments (Experiment 1). Developmental stages are from fertilization to eyed egg (left), eyed egg to start feed (middle), and from start feed to parr (right). Data from all three half-sibling families are pooled.

In Experiment 1, mortality rates were lowest for the 0 PSI treatment (1.7% from fertilization until the eyed egg stage, 3% from eyed egg to start-feeding, and 3.8% from start-feeding to the parr stage), somewhat higher in the 9500 PSI treatment (5.4% fertilization to eyed egg, 5.7% eyed egg to start-feeding, and 6.2% start-feeding to parr), and highest at the intermediate pressure treatments (e.g., 6.3% fertilization to eyed egg, 31.7% eyed egg to start-feeding, and 13.4% start-feeding to parr in the 7500 PSI group). Mortality rates were highest between the eyed egg stage and first feeding (Fig. 5). Mortality rates by family, available only from fertilization until the eyed egg stage, showed the highest mortality for family 1 (ranging 3.7% at 0 PSI to 19.8% at 8500 PSI) and the lowest mortality for family 3 (<1.4% across all treatments, Supplementary Fig. 6).

**Fig. 6:**
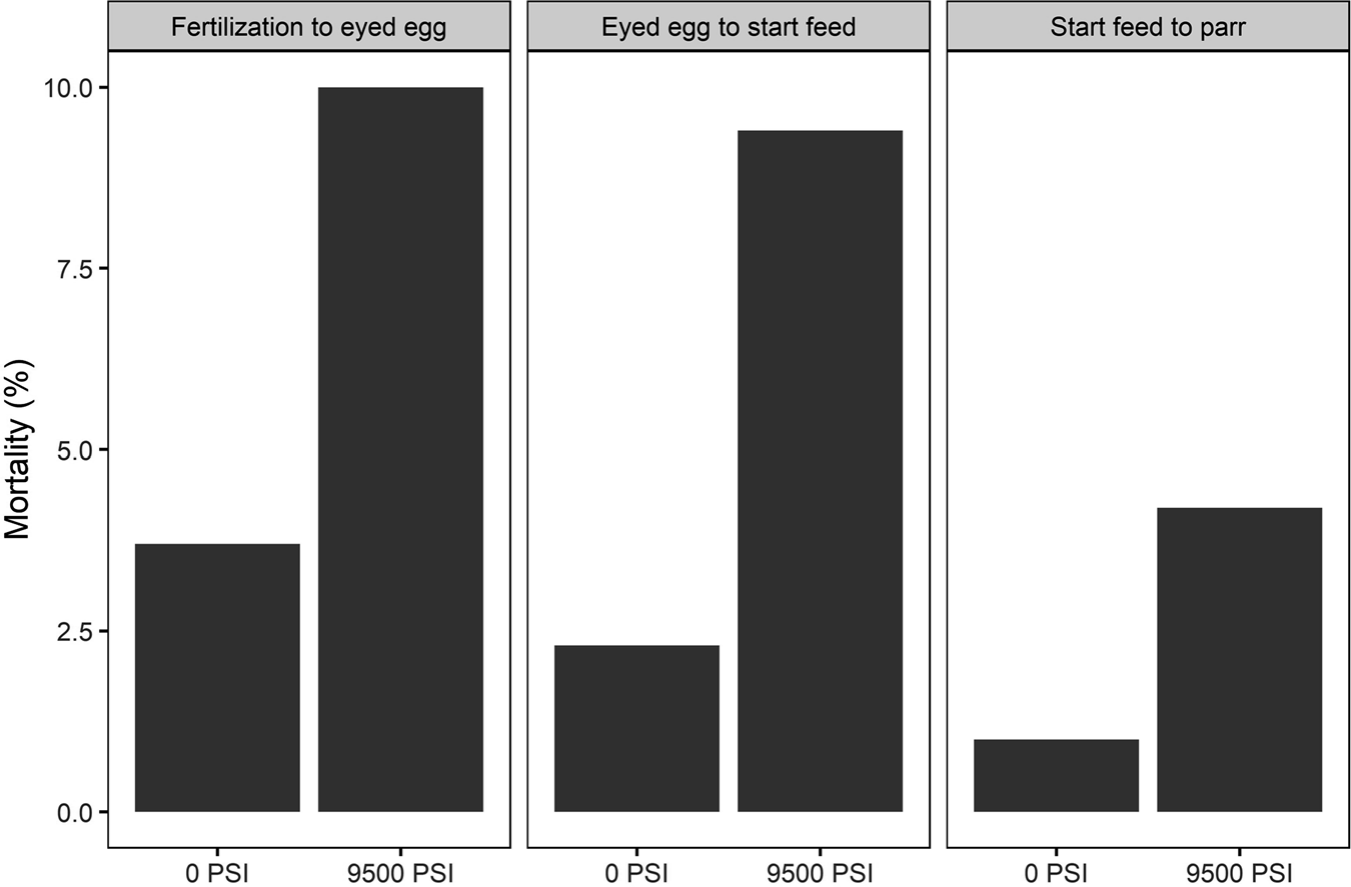
Mortality rates during early life stages following standard diploid and triploid hydrostatic pressure treatments (Experiment 1). Developmental stages are from fertilization to eyed egg (left), eyed egg to start feed (middle) and from start feed to parr (right). Data from all 12 half-sibling families are pooled.

In Experiment 2, mortality rates were higher in the 9500 PSI treatment than in the 0 PSI treatment at every developmental stage (10% and 3.7% from fertilization until the eyed egg stage, 9.4% and 2.3% from eyed egg to start feeding, and 4.2% and 1% from start-feeding to parr, respectively, Fig. 6). Higher mortality in the 9500 PSI treatment was consistent across most families between fertilization and the eyed egg stage, although there was a large variation in mortality rates among families (Supplementary Fig. 7).

## Discussion

We have investigated the incidence of chromosomal aberrations among Atlantic salmon subjected to a gradient of hydrostatic pressure treatments shortly after fertilization, simulating potential inefficiencies in a standard procedure used to produce sterile triploid fish in the aquaculture industry. Building on the findings of Glover et al. (2020), who first documented aberrant inheritance in pressure-induced triploid Atlantic salmon, our results revealed that while chromosomal aberrations can occur among both diploid (0 PSI) and pressure-induced triploids (9500 PSI), sub-optimal pressure treatments (6500–8500 PSI) increase the incidence of chromosomal aberrations above this background rate. A family effect across both experiments further suggested that the maternal germline may also play a role. Chromosomal aberrations were common at the eyed egg stage but infrequent at the parr stage, suggesting that maladapted individuals were purged during early development. To our knowledge, this study is the first to quantitatively investigate chromosomal aberrations to this level of detail in a teleost fish, as most studies to date have relied on cytological (e.g., Glover et al. 2015; Preston et al. 2013; Stien et al. 2019) or qualitative genetic (e.g., Glover et al. 2020; Jacq 2021; Sanz et al. 2020) assessments of ploidy.

Our results demonstrate that triploidization success decreases when the hydrostatic pressure decreases below the industry standard of 9500 PSI, and this was most noticeable below 8500 PSI (Experiment 1). This is in line with experiments performed to optimize hydrostatic pressure treatments to produce triploids in other species (Káldy et al. 2021; Preston et al. 2013), and implies that, below a certain threshold, the hydrostatic pressure becomes insufficient to retain the second polar body in all eggs during meiosis II. To date, most studies reporting the occurrence of “failed triploids” following hydrostatic or thermal shock treatments have used cytological or qualitative microsatellite assessments and assumed that the remaining non-triploid fish were diploids (Glover et al. 2015; Preston et al. 2013; Stien et al. 2019). However, by assessing the somy at each of ~20 microsatellite loci using allelic dosage information (MAC-PR, Delaval et al. 2024), our results suggest that some of these “failed triploids” may in fact be aneuploids and occasionally exhibit additional forms of chromosomal aberrations, in particular a failure to inherit maternal genetic material. Although we did not detect it in our experiment, earlier studies have shown that a failure to inherit paternal genetic material can also occur (Devlin et al. 2010; Glover et al. 2020). The occurrence of aneuploids and mosaics following hydrostatic pressure treatments has long been known, although this mostly comes from studies that applied the pressure treatment to inhibit mitotic (rather than meiotic) cleavage to produce tetraploids (Yamazaki and Goodier 1993; Zhang and Onozato 2004). Our results support the findings of a recent study documenting a similar phenomenon in pikeperch (*Sander lucioperca*) subjected to hydrostatic pressure to inhibit meiotic cleavage (Káldy et al. 2021).

Among Atlantic salmon produced using standard diploid (no hydrostatic pressure) and triploid (9500 PSI) treatments, aneuploidy was detected among 10–17% of eyed eggs, reducing to <2% by the parr stage. A few individuals (<1%) from both the diploid and triploid treatments also displayed incomplete inheritance of maternal alleles, which is in line with the findings of Glover et al. (2020), although that study did not detect any aberrations among diploid fish. It is not clear whether aneuploidy and other chromosomal aberrations occur naturally in Atlantic salmon or whether this is an effect of domestication, and this would require a comparison to wild samples. However, the rates of aneuploidy observed are comparable to literature on other vertebrates, which is currently mostly limited to humans (Taylor et al. 2014; Webster and Schuh 2017) and domesticated mammals (Schmutz et al. 1996; Shilton et al. 2020). In these studies, as in ours, aneuploidy was seldom detected among surviving offspring, suggesting that this condition is generally lethal in early development. The relatively few aneuploid parr that survived tended to be individuals with only one or two trisomies, suggesting that certain trisomies are viable. The production of unviable aneuploid eggs that do not survive long beyond the eyed egg stage may therefore be a natural phenomenon in fish, but this requires further investigation.

Chromosomal aberrations occurred at higher frequencies following sub-optimal hydrostatic pressure treatments. The highest incidence of aneuploidy was detected among eyed eggs subjected to 6500–7500 PSI (35–38% of eyed eggs), and while incomplete inheritance of maternal DNA was detected in all treatments across both experiments, the incidence of these aberrations was highest across the three sub-optimal pressure treatments (6500–8500 PSI, Experiment 1). This correlated with higher mortality rates in these treatments, particularly between the eyed egg stage and start feeding, indicating that cellular or organismal fitness may have been compromised in these individuals (Williams et al. 2008). We can only hypothesize as to the mechanisms causing chromosomal aberrations in Atlantic salmon. There are multiple precise cellular processes involved in the correct alignment and segregation of homologous chromosomes during cell division (Orr et al. 2015; Taylor et al. 2014), and deviations to either of these processes may lead to nondisjunction.

The family effect that we observed suggests an effect of the maternal germline on the incidence of these meiotic errors, which supports similar maternal effects detected in other vertebrates (Rizzo et al. 2019; Taylor et al. 2014; Thomas et al. 2021; Webster and Schuh 2017). One hypothesis is that the optimal timing of the hydrostatic pressure treatment may vary depending on the rate of embryonic development, which can be affected by variables such as temperature and egg quality (Galbreath and Samples 2000; Preston et al. 2013), and that deviations from optimal timings may result in chromosomal aberrations. The evidence here is mixed, with some studies suggesting that changes in the timing of thermal/pressure shocks (within the appropriate cell division phase window) have little effect on the success of ploidy manipulation (Galbreath and Samples 2000; Preston et al. 2013), and others demonstrating an effect (Galbreath and Samples 2000; Hershberger and Hostuttler 2007). The exact mechanisms behind these chromosomal aberrations are unclear. However, our results demonstrate that both the efficiency of hydrostatic pressure treatments and maternal variables can affect the incidence of chromosomal aberrations in Atlantic salmon.

On rare occasions, genetic material may only be inherited from one parent and result in uniparental disomy (UPD), a phenomenon that has been observed in humans where it has pathogenic consequences (Benn 2021; Taylor et al. 2014), and has also been detected previously in pressure-induced triploid salmon (Glover et al. 2020). UPD can be explained by a genetic rescue event whereby a meiotic error is corrected for in a later mitotic event, reverting daughter cells back from a trisomic to a disomic state (Benn 2021; Taylor et al. 2014). In the present study, 12 cases of UPD were identified, where the individuals lacked maternal alleles at one or several loci. Eleven of these cases were observed in eyed eggs, eight of which were the products of sub-optimal pressure treatments (Experiment 1). Only one surviving parr exhibited UPD (Experiment 2), however this individual had very slow growth and displayed signs of poor welfare (qualitative observation), strongly suggesting a pathogenic effect. In five of these cases (four eyed eggs and the aforementioned parr), the individuals displayed total or near-total homozygosity. While our genetic protocol was unable to resolve ploidy in homozygotic cases, it is possible that these individuals exhibited androgenetic haploidy or double-haploidy, a phenomenon that has been observed previously in pressure and temperature shocked progeny (see Morishima et al. 2011).

Interestingly, individuals inheriting two paternal alleles at a locus were only detected at low hydrostatic pressures (0 PSI and 6500 PSI). In these treatments, the trisomies detected were either the result of inheriting two maternal alleles, two paternal alleles, or inheriting an extra allele shared by both parents where its origin was ambiguous. Therefore, it appears that in the absence of sufficient hydrostatic pressure, spontaneous trisomies may either derive from aneuploid oocytes or sperm or nondisjunction. This also indicates that the hydrostatic pressure treatment, when strong enough, is highly effective at preventing paternally derived trisomies. The fact that offspring subjected to hydrostatic pressure occasionally lacked maternal rather than paternal alleles indicates that chromosomal aberrations are mostly brought on by a failure of the pressure treatment to retain all genetic material from the second polar body.

Previously, evaluations of ploidy using microsatellite markers were based on qualitative assessments of electropherograms, with triploids identified by the presence of three distinct alleles at one or several loci (Glover et al. 2020). By using MAC-PR (Delaval et al. 2024), which considers allele dosage or relative copy number when two allele variants are present, we were able to access somy information across all loci exhibiting a heterozygous genotype and infer the parental origin of each copy. However, the main limitation of using microsatellites remains homozygous loci, since allele copy number and therefore somy cannot be determined from common techniques using PCR and fragment analysis. In addition, parents sometimes share alleles or differ by few repeat units, meaning that the parental origin of individual allele copies was sometimes ambiguous, particularly if single repeat motif shift mutations had occurred. Finally, we only considered 20 microsatellites as informative in this study, which is an underestimation of genome-wide inheritance patterns and does not even cover all the Atlantic salmon’s 29 chromosomes (Lien et al. 2016, Supplementary Table S1). These considerations imply that our data represent minimum estimates. Nonetheless, our microsatellite-based approach was practical and cost-effective, allowing us to investigate this phenomenon across several thousand eggs and parr. It also represents an effective tool that can be applied for the verification of ploidy and chromosomal aberrations in commercial settings. Our findings may warrant a more detailed investigation into the abovementioned phenomena using higher resolution genome-wide approaches.

### Practical implications and future perspectives

In the aquaculture industry, producing sterile fish is an important part of the solution to prevent farm-to-wild introgression following escape events (Benfey 2016; Glover et al. 2017; Piferrer et al. 2009). Experimental and commercial trials have shown that producing sterile triploid Atlantic salmon can be challenging, as they occasionally suffer from poor welfare and high mortality rates (Fraser et al. 2012; Madaro et al. 2022; Stien et al. 2019). Although new strategies are being proposed to induce sterility (Andersen et al. 2022; Güralp et al. 2020; Kleppe et al. 2020; Wargelius et al. 2016), the production of triploids using hydrostatic pressure treatments is currently the most practical and scalable solution. Our study has shown that if the hydrostatic pressure treatment is not applied efficiently, it could lead to higher numbers of offspring with chromosomal aberrations and may contribute to higher mortality in early development. Not only would this represent an economic loss to the industry, but a failure to induce triploidy also jeopardizes its use as a biocontainment strategy. Furthermore, the variation in chromosomal aberrations detected among half-sibling families suggests that breeding programs should consider variables relating to the maternal germline when selecting crosses to produce pressure-induced triploids. Whether or not the types of chromosomal aberrations observed contribute to poor welfare in surviving fish should now be the subject of investigation. This could be achieved by genotyping losses resulting from unexplained poor welfare from commercial farms, or by scaling up the present experiment and evaluating the welfare of surviving fish for growth, deformities, and pathologies.

Our study may be among the first to document a link between chromosomal aberrations and mortality in early development in a teleost fish. It would be interesting to investigate the extent to which this phenomenon occurs, not only in farm and aquaculture settings where this may represent an avenue to improve production and welfare, but also in wild populations.

## Supplementary information

Supplementary material

## Data Availability

The data associated with this study have been deposited in a public Dryad repository with the following doi: 10.5061/dryad.4xgxd25jx. These include the genotypes of all offspring from experiments 1 and 2 (resolved using MAC-PR), the genotypes of their parents, summary statistics, and detailed genotypes of offspring displaying inheritance aberrations.
