# Supplementary material for: Chromosomal aberrations and early mortality in a non-mammalian vertebrate: example from pressure-induced triploid Atlantic salmon
Source: Heredity (Edinb). 2024 Oct 5;133(6):426–36. doi: 10.1038/s41437-024-00727-9 (PMC11589116; doi:10.1038/s41437-024-00727-9)
Supplement: Supplementary file 1 — Supplementary material [file 41437_2024_727_MOESM1_ESM.docx]

**Supplementary File: Chromosomal aberrations and early mortality in a non-mammalian vertebrate: example from pressure-induced triploid Atlantic salmon**

*Table S1: 30 Atlantic salmon* (Salmo salar) *microsatellite markers used in this study. BLAST searches of primer sequences from the original publications were performed against the latest* S. salar *assembly (Ssal3.1) to retrieve their chromosomal position, where available. ‘NA’ refers to ambiguous or low quality hits.*

| Locus name | Reference | BLAST |
| --- | --- | --- |
| Multiplex 1 |  |  |
| SSsp2210 | Paterson, S. et al. (2004). Characterization and PCR multiplexing of novel highly variable tetranucleotide Atlantic salmon (*Salmo salar* L.) microsatellites. *Molecular Ecology Notes*, 4(2), 160-162. | SSA12 |
| SSspG7 | Paterson, S. et al. (2004). Characterization and PCR multiplexing of novel highly variable tetranucleotide Atlantic salmon (*Salmo salar* L.) microsatellites. *Molecular Ecology Notes*, 4(2), 160-162. | NA |
| SsaD144 | King, T. L. et al. (2005). Microsatellite DNA markers for the study of Atlantic salmon (*Salmo salar*) kinship, population structure, and mixed‐fishery analyses. *Molecular Ecology Notes*, 5(1), 130-132. | SSA03 |
| Ssa202 | O'Reilly, P. T. et al. (1996). Rapid analysis of genetic variation in Atlantic salmon (Salmo salar) by PCR multiplexing of dinucleotide and tetranucleotide microsatellites. *Canadian Journal of Fisheries and Aquatic Sciences*, 53(10), 2292-2298. | SSA09/SSA02 |
| Sp2201 | Paterson, S. et al. (2004). Characterization and PCR multiplexing of novel highly variable tetranucleotide Atlantic salmon (*Salmo salar* L.) microsatellites. *Molecular Ecology Notes*, 4(2), 160-162. | SSA13 |
| SsaD157 | King, T. L. et al. (2005). Microsatellite DNA markers for the study of Atlantic salmon (*Salmo salar*) kinship, population structure, and mixed‐fishery analyses. *Molecular Ecology Notes*, 5(1), 130-132. | SSA26/SSA02 |
| Multiplex 2 |  |  |
| Ssa289 | McConnell, S. K. et al. (1995). Polymorphic microsatellite loci from Atlantic salmon (*Salmo salar*): genetic differentiation of North American and European populations. *Canadian Journal of Fisheries and Aquatic Sciences*, 52(9), 1863-1872. | SSA19/SSA16/ SSA14 |
| Ssa14 | McConnell, S. K. et al. (1995). Polymorphic microsatellite loci from Atlantic salmon (*Salmo salar*): genetic differentiation of North American and European populations. *Canadian Journal of Fisheries and Aquatic Sciences*, 52(9), 1863-1872. | NA |
| Sp1605 | Paterson, S. et al. (2004). Characterization and PCR multiplexing of novel highly variable tetranucleotide Atlantic salmon (*Salmo salar* L.) microsatellites. *Molecular Ecology Notes*, 4(2), 160-162. | SSA23 |
| Ssa171 | O'Reilly, P. T. et al. (1996). Rapid analysis of genetic variation in Atlantic salmon (Salmo salar) by PCR multiplexing of dinucleotide and tetranucleotide microsatellites. *Canadian Journal of Fisheries and Aquatic Sciences*, 53(10), 2292-2298. | SSA06/SSA03 |
| Sp2216 | Paterson, S. et al. (2004). Characterization and PCR multiplexing of novel highly variable tetranucleotide Atlantic salmon (*Salmo salar* L.) microsatellites. *Molecular Ecology Notes*, 4(2), 160-162. | SSA24 |

*Table S1 (continued)*

| Locus name | Reference | BLAST |
| --- | --- | --- |
| Multiplex 3 |  |  |
| SsaF43 | Sanchez, J. A. et al. (1996). Protein and microsatellite single locus variability in *Salmo salar* L. (Atlantic salmon). *Heredity*, 77(4), 423-432. | SSA16 |
| Ssa197 | O'Reilly, P. T. et al. (1996). Rapid analysis of genetic variation in Atlantic salmon (Salmo salar) by PCR multiplexing of dinucleotide and tetranucleotide microsatellites. *Canadian Journal of Fisheries and Aquatic Sciences*, 53(10), 2292-2298. | SSA15 |
| SSsp3016 | Genbank AY372820 | SSA11 |
| MHC1 | Grimholt, U. et al. (2002). The major histocompatibility class I locus in Atlantic salmon (*Salmo salar* L.): polymorphism, linkage analysis and protein modelling. *Immunogenetics*, 54, 570-581. | NA |
| MHC2 | Stet, R. J. et al. (2002). Unique haplotypes of co-segregating major histocompatibility class II A and class II B alleles in Atlantic salmon (*Salmo salar*) give rise to diverse class II genotypes. *Immunogenetics*, 54, 320-331. | NA |
| SsOSL85 | Slettan, A. et al. (1995). Atlantic salmon, *Salmo salar*, microsatellites at the SSOSL25, SSOSL85, SSOSL311, SSOSL417 loci. *Animal Genetics*, 26(4), 281-282. | NA |
|  |  |  |
| Multiplex 4 |  |  |
| Ssa412 | Cairney, M. et al. (2000). Characterization of microsatellite and minisatellite loci in Atlantic salmon (*Salmo salar* L.) and cross-species amplification in other salmonids. *Molecular Ecology*, 9(12), 2175-2178. | SSA09 |
| Ssa405 | Cairney, M. et al. (2000). Characterization of microsatellite and minisatellite loci in Atlantic salmon (*Salmo salar* L.) and cross-species amplification in other salmonids. *Molecular Ecology*, 9(12), 2175-2178. | SSA04 |
| Ssa98 | Genbank AF019195 | SSA15 |
| SsOSL25 | Slettan, A. et al. (1995). Atlantic salmon, *Salmo salar*, microsatellites at the SSOSL25, SSOSL85, SSOSL311, SSOSL417 loci. *Animal Genetics*, 26(4), 281-282. | SSA19/SSA14 |
| SSsp2215 | Paterson, S. et al. (2004). Characterization and PCR multiplexing of novel highly variable tetranucleotide Atlantic salmon (*Salmo salar* L.) microsatellites. *Molecular Ecology Notes*, 4(2), 160-162. | SSA24 |
| EST107 | Vasemägi, A. et al. (2005). Seventy‐five EST‐linked Atlantic salmon (*Salmo salar* L.) microsatellite markers and their cross‐amplification in five salmonid species. *Molecular Ecology Notes*, 5(2), 282-288. | NA |
| EST68 | Vasemägi, A. et al. (2005). Seventy‐five EST‐linked Atlantic salmon (*Salmo salar* L.) microsatellite markers and their cross‐amplification in five salmonid species. *Molecular Ecology Notes*, 5(2), 282-288. | NA |
| Multiplex 5 |  |  |
| EST28 | Vasemägi, A. et al. (2005). Seventy‐five EST‐linked Atlantic salmon (*Salmo salar* L.) microsatellite markers and their cross‐amplification in five salmonid species. *Molecular Ecology Notes*, 5(2), 282-288. | NA |
| EST19 | Vasemägi, A. et al. (2005). Seventy‐five EST‐linked Atlantic salmon (*Salmo salar* L.) microsatellite markers and their cross‐amplification in five salmonid species. *Molecular Ecology Notes*, 5(2), 282-288. | NA |
| Ssa407 | Cairney, M. et al. (2000). Characterization of microsatellite and minisatellite loci in Atlantic salmon (*Salmo salar* L.) and cross-species amplification in other salmonids. *Molecular Ecology*, 9(12), 2175-2178. | NA |
| Ssleer15.1 | Genbank U86708 | SSA20/SSA24 |
| Sleen82 | Genbank U86706 | SSA04 |
| Sleer53 | Genbank U86704 | SSA03 |

*Table S2: PCR reactions for each of five multiplexes*

| **Promul 1** | **Concentration (stock)** | **Concentration**  **(per reaction)** | **Volume**  **(per reaction)** |
| --- | --- | --- | --- |
| **DNA** |  | ~ 33 ng | 2 µL |
| **Buffer** | 5 x | 1 x | 2 µL |
| **MgCl_2_** | 25 mM | 2 mM | 0.8 µL |
| **dNTP** | 1.25 mM | 0.2 mM | 1.6 µL |
| **GoTaq G2 pol** | 5 U | 0.35 U | 0.07 µL |
| **SSsp2210-F/R** | 10 mM | 0.08 mM | 0.08 µL |
| **SSspG7-F/R** | 10 mM | 0.15 mM | 0.15 µL |
| **SsaD144-F/R** | 10 mM | 0.23 mM | 0.23 µL |
| **Ssa202-F/R** | 10 mM | 0.08 mM | 0.08 µL |
| **Sp2201-F/R** | 10 mM | 0.25 mM | 0.25 µL |
| **SsaD157-F/R** | 10 mM | 0.25 mM | 0.25 µL |
| **H_2_O** |  |  | 2.49 µL |
| **Total** |  |  | 10 µL |
|  |  |  |  |
| **Promul 2** | **Concentration (stock)** | **Concentration**  **(per reaction)** | **Volume**  **(per reaction)** |
| **DNA** |  | ~ 33 ng | 2 µL |
| **Buffer** | 5 x | 1 x | 2 µL |
| **MgCl_2_** | 25 mM | 2 mM | 0.8 µL |
| **dNTP** | 1.25 mM | 0.2 mM | 1.6 µL |
| **GoTaq G2 pol** | 5 U | 0.35 U | 0.07 µL |
| **Ssa289 F/R** | 10 mM | 0.4 mM | 0.4 µL |
| **Ssa14 F/R** | 10 mM | 0.12 mM | 0.12 µL |
| **Sp1605 F/R** | 10 mM | 0.1 mM | 0.1 µL |
| **Ssa171 F/R** | 10 mM | 0.2 mM | 0.2 µL |
| **Sp2216 F/R** | 10 mM | 0.015 mM | 0.015 µL |
| **Exon2_F/R*** | 10 mM | 0.05 mM | 0.05 µL |
| **Exon4_F/R*** | 10 mM | 0.05 mM | 0.05 µL |
| **H_2_O** |  |  | 2.595 µL |
| **Total** |  |  | 10 µL |
| *Sex marker part of primer mix, but not used in analyses | | | |

*Table S2 (continued)*

| **Promul 3** | **Concentration (stock)** | **Concentration**  **(per reaction)** | **Volume**  **(per reaction)** |
| --- | --- | --- | --- |
| **DNA** |  | ~ 33 ng | 2.00 µL |
| **Buffer** | 5 x | 1 x | 2.00 µL |
| **MgCl_2_** | 25 mM | 2 mM | 0.80 µL |
| **dNTP** | 1.25 mM | 0.2 mM | 1.60 µL |
| **GoTaq G2 pol** | 5 U | 0.35 U | 0.07 µL |
| **SsaF43 F/R** | 10 mM | 0.05 mM | 0.05 µL |
| **Ssa197 F/R** | 10 mM | 0.05 mM | 0.05 µL |
| **SsaD486 F/R** | 10 mM | 0.05 mM | 0.05 µL |
| **SSsp3016 F/R** | 10 mM | 0.1 mM | 0.10 µL |
| **MHC1 F/R** | 10 mM | 0.05 mM | 0.05 µL |
| **MHC2 F/R** | 10 mM | 0.12 mM | 0.12 µL |
| **SsOSL85 F/R** | 10 mM | 0.23 mM | 0.23 µL |
| **H_2_O** |  |  | 2.88 µL |
| **Total** |  |  | 10.00 µL |
|  |  |  |  |
| **KAPA 1** | **Concentration (stock)** | **Concentration**  **(per reaction)** | **Volume**  **(per reaction)** |
| **DNA** |  | ~ 25 ng | 2.000 µL |
| **KAPA2G™ Fast HotStart ready mix (2x)** |  |  | 4.000 µL |
| **Ssa412** | 50 mM | 0.192 mM | 0.031 µL |
| **Ssa405** | 50 mM | 0.385 mM | 0.062 µL |
| **Ssa98** | 50 mM | 0.160 mM | 0.026 µL |
| **SsOSL25** | 50 mM | 0.192 mM | 0.031 µL |
| **SSsp2215** | 50 mM | 0.128 mM | 0.020 µL |
| **EST107** | 50 mM | 0.500 mM | 0.080 µL |
| **EST68** | 50 mM | 0.200 mM | 0.032 µL |
| **H_2_O** |  |  | 1.719 µL |
| **Total** |  |  | 8.000 µL |
|  |  |  |  |
| **KAPA 2** | **Concentration (stock)** | **Concentration**  **(per reaction)** | **Volume**  **(per reaction)** |
| **DNA** |  | ~ 25 ng | 2.000 µL |
| **KAPA2G™ Fast HotStart ready mix (2x)** |  |  | 4.000 µL |
| **EST28** | 50 mM | 0.190 mM | 0.030 µL |
| **EST19** | 50 mM | 0.241 mM | 0.039 µL |
| **Ssa407** | 50 mM | 0.474 mM | 0.076 µL |
| **Ssleer15.1** | 50 mM | 0.150 mM | 0.024 µL |
| **Sleen82** | 50 mM | 0.353 mM | 0.056 µL |
| **Sleer53** | 50 mM | 0.200 mM | 0.032 µL |
| **H_2_O** |  |  | 1.743 µL |
| **Total** |  |  | 8.000 µL |

*Table S3: PCR thermal cycle settings for five multiplexes*

| **Promul 1, Promul 2 & Promul 3** | | | |
| --- | --- | --- | --- |
| **Step** | **Temperature (°C)** | **Time** | **Cycles** |
| 1 | 94 | 4 min | 1 |
| 2 | 94 | 50 s | 33 |
| 3 | 55 | 50 s |  |
| 4 | 72 | 80 s |  |
| 5 | 72 | 10 min | 1 |
| 6 | 4 | ∞ | 1 |
|  |  |  |  |
| **KAPA 1** |  |  |  |
| **Step** | **Temperature (°C)** | **Time** | **Cycles** |
| 1 | 95 | 150 | 1 |
| 2 | 95 | 25 | 9 |
| 3 | 58 | 30 |  |
| 4 | 72 | 25 |  |
| 5 | 95 | 25 | 20 |
| 6 | 53 | 30 |  |
| 7 | 72 | 25 |  |
| 8 | 72 | 600 | 1 |
| 9 | 12 | 120 | 1 |
| 10 | 4 | ∞ | 1 |
|  |  |  |  |
| **KAPA 2** |  |  |  |
| **Step** | **Temperature (°C)** | **Time** | **Cycles** |
| 1 | 95 | 150 | 1 |
| 2 | 95 | 25 | 9 |
| 3 | 58 | 30 |  |
| 4 | 72 | 25 |  |
| 5 | 95 | 25 | 18 |
| 6 | 53 | 30 |  |
| 7 | 72 | 25 |  |
| 8 | 72 | 600 | 1 |
| 9 | 12 | 120 | 1 |
| 10 | 4 | ∞ | 1 |

Table S4: Results and model selection of the Generalized Linear Mixed Models (Experiment 1) and Generalized Linear Models (Experiment 2), testing the incidence of triploids and aneuploids across hydrostatic pressure treatments, families, and developmental stages (eyed egg or parr). ΔAIC reports the difference in AIC when compared to the retained model (in bold).

| **Experiment 1** | | | | | | | | | |
| --- | --- | --- | --- | --- | --- | --- | --- | --- | --- |
| **Sample size** | |  | **Fixed effects** | | | **Random effects** |  |  | |
| **Egg** | **Parr** | **Response** | **Pressure** | **Family** | **Stage** | **Replicate** | **AIC** | | **ΔAIC** |
| 437 | 1,098 | Triploid* | **x** | **x** | **x** | **x** | **459.7** | |  |
|  |  |  |  | x | x | x | 485.6 | | 25.9 |
|  |  |  | x |  | x | x | 642.6 | | 182.9 |
|  |  |  | x | x |  | x | 533.3 | | 73.6 |
|  |  |  |  |  |  |  |  | |  |
|  |  | Aneuploid | **x** | **x** | **x** | **x** | **663.2** | |  |
|  |  |  |  | x | x | x | 672.2 | | 9.0 |
|  |  |  | x |  | x | x | 685.0 | | 21.8 |
|  |  |  | x | x |  | x | 811.5 | | 148.3 |
|  | | | | | | | | | |
| **Experiment 2** | | | | | | | | | |
| **Sample size** | |  | **Fixed effects** | | |  |  |  | |
| **Egg** | **Parr** | **Response** | **Pressure** | **Family** | **Stage** |  | **AIC** | | **ΔAIC** |
| 470 | 2,179 | Triploid | **x** | **x** | **x** |  | **348.3** | |  |
|  |  |  |  | x | x |  | 3637.2 | | 3288.9 |
|  |  |  | x |  | x |  | 385.2 | | 36.9 |
|  |  |  | x | x |  |  | 399.1 | | 50.8 |
|  |  |  |  |  |  |  |  | |  |
|  |  | Aneuploid | **x** | **x** | **x** |  | **567.4** | |  |
|  |  |  |  | x | x |  | 571.5 | | 4.1 |
|  |  |  | x |  | x |  | 616.6 | | 49.2 |
|  |  |  | x | x |  |  | 678.3 | | 110.9 |

* Model convergence problem in the initial model due to quasi-complete separation. This model was run after dropping the 0 PSI pressure treatment.


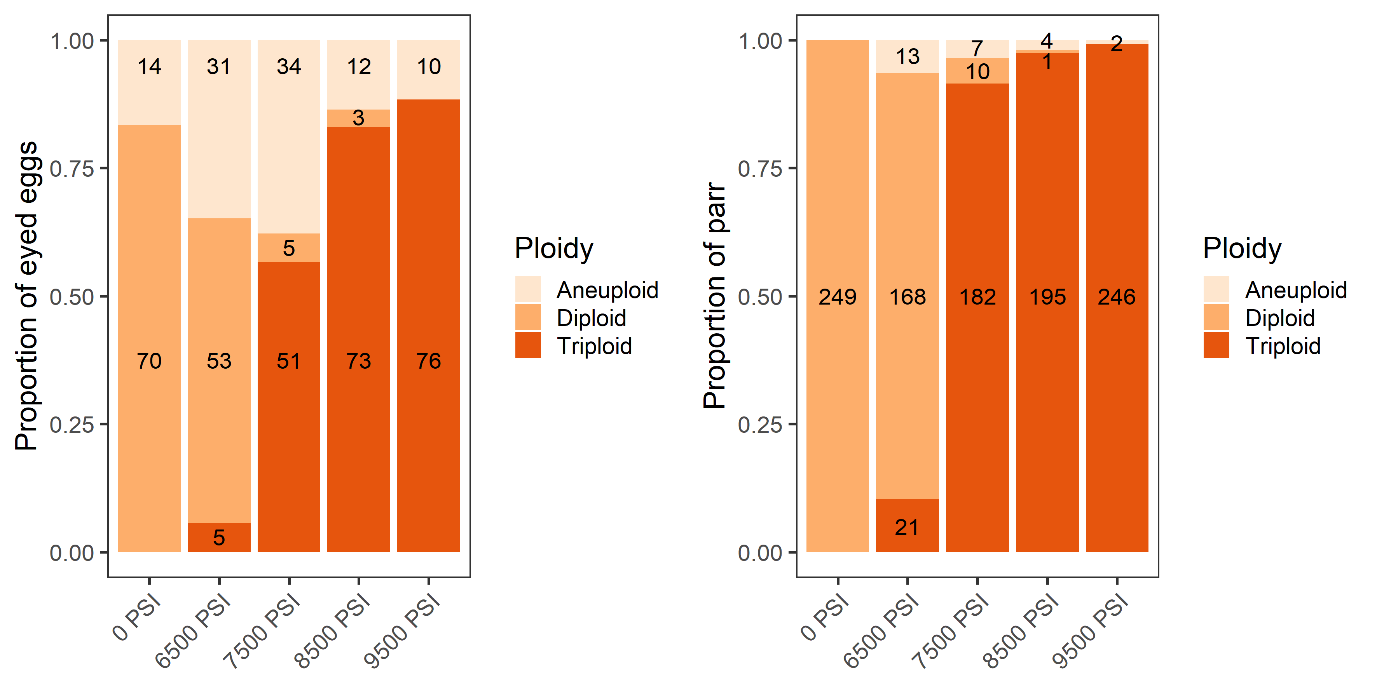


*Supplementary Figure 1: The proportion of eyed eggs (N=437) and surviving parr (N=1,098) from three families (pooled) subjected to five hydrostatic pressure treatments in Experiment 1 categorised as diploids, triploids, or aneuploids based on microsatellite analysis. Numbers of individuals in each group are shown.*


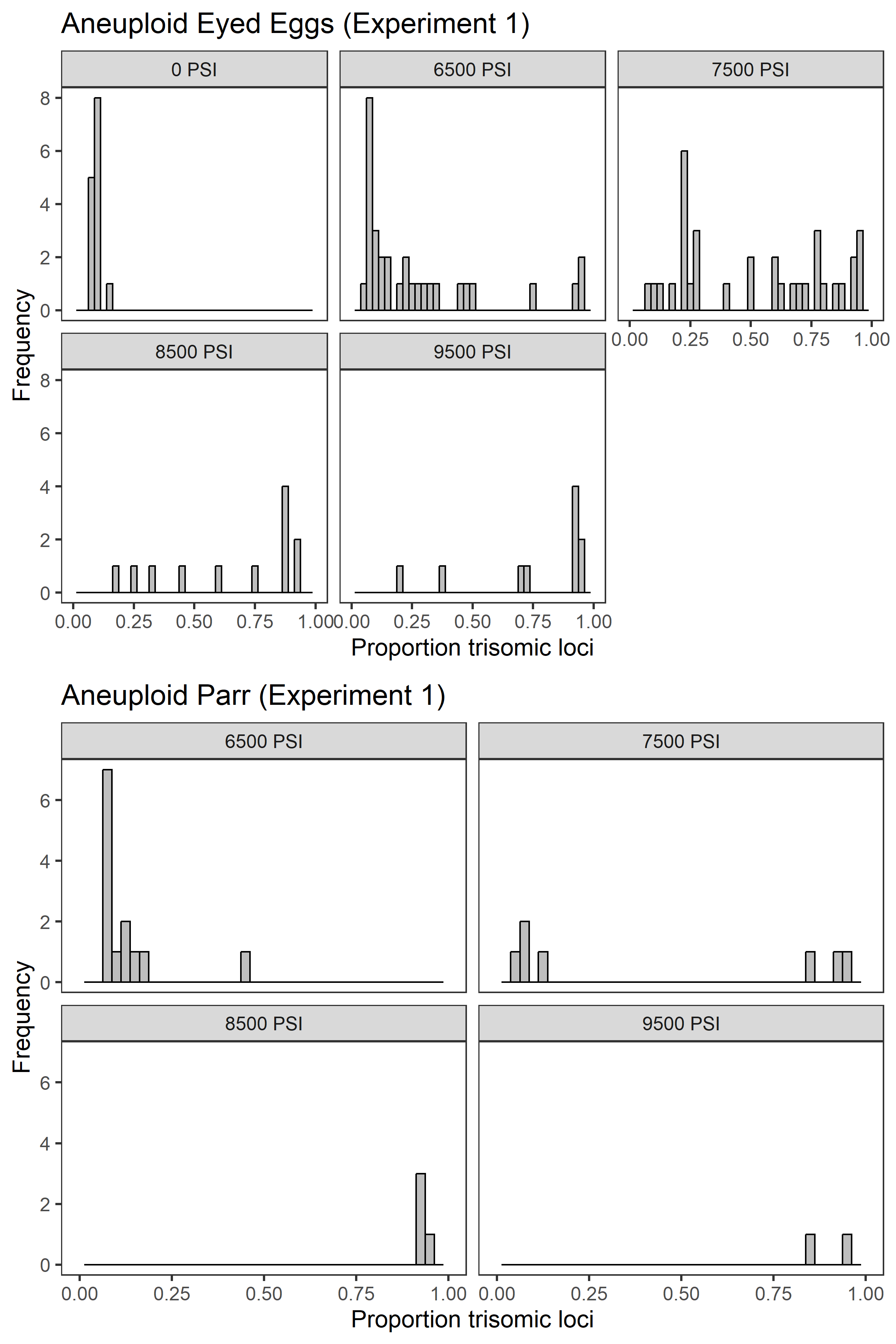


*Supplementary Figure 2: Proportion of heterozygotic loci displaying trisomy among aneuploid eyed eggs (top) subjected to five hydrostatic pressure treatments (Experiment 1), and among surviving aneuploid parr (bottom).*


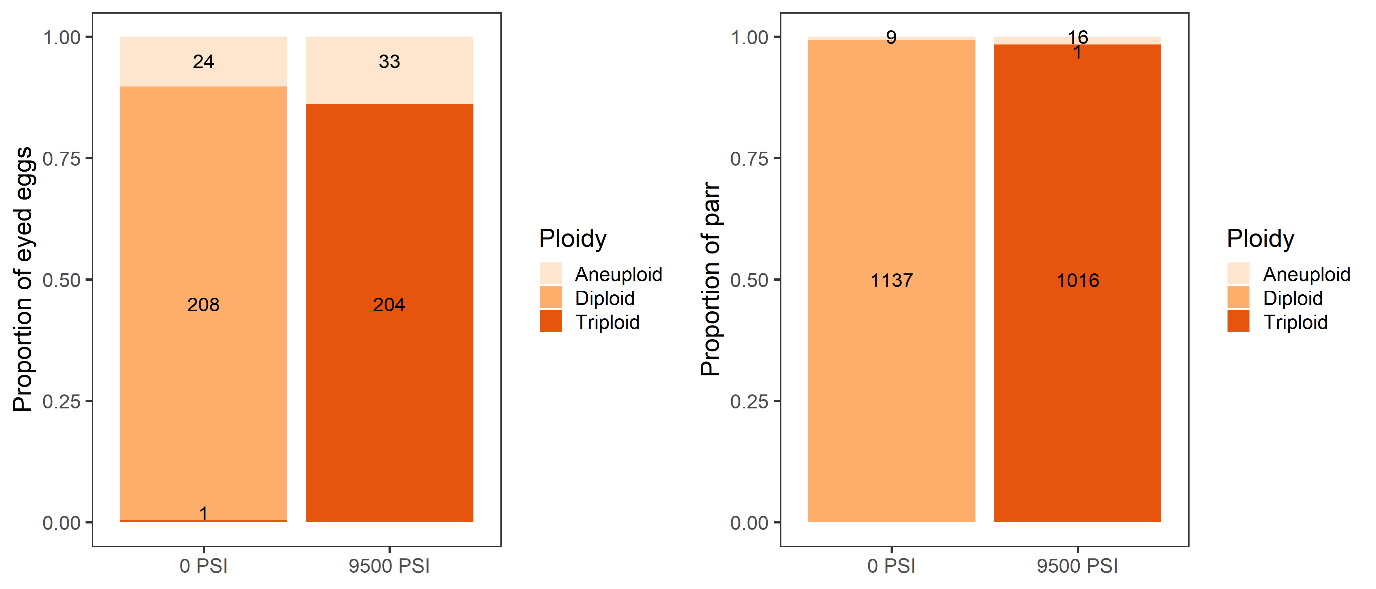


*Supplementary Figure 3: The proportion of eyed eggs (N=470) and surviving parr (N=2,179) from 12 families (pooled) subjected to standard diploid (0 PSI) and triploid (9500 PSI) pressure treatments in Experiment 2 categorised as diploids, triploids, or aneuploids based on microsatellite analysis. Numbers of individuals in each group are shown.*


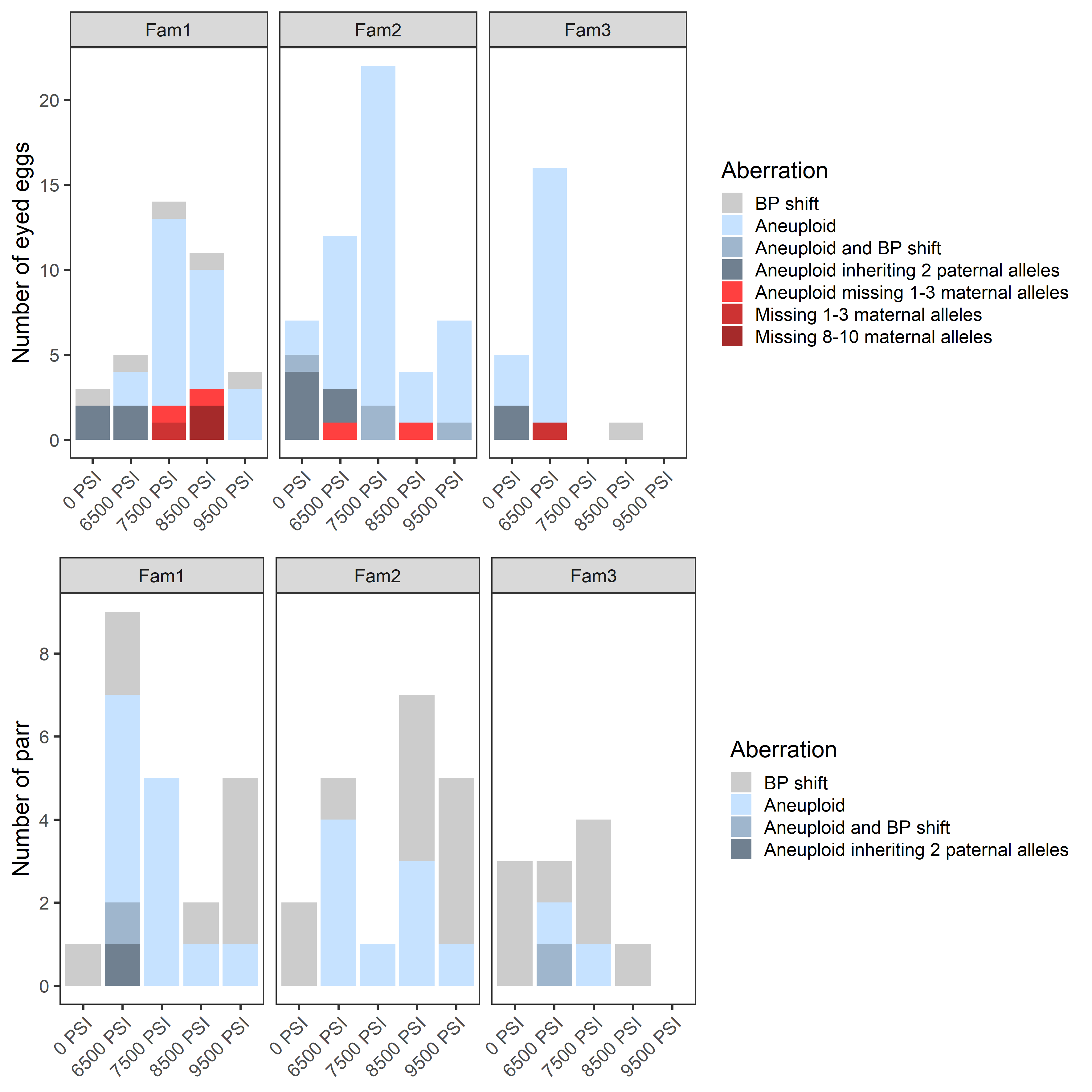


*Supplementary Figure 4: Incidence of inheritance aberrations among eyed eggs (top, N=438) and surviving parr (bottom, N=1,098) subjected to 5 hydrostatic pressure treatments (Experiment 1), grouped by family.*


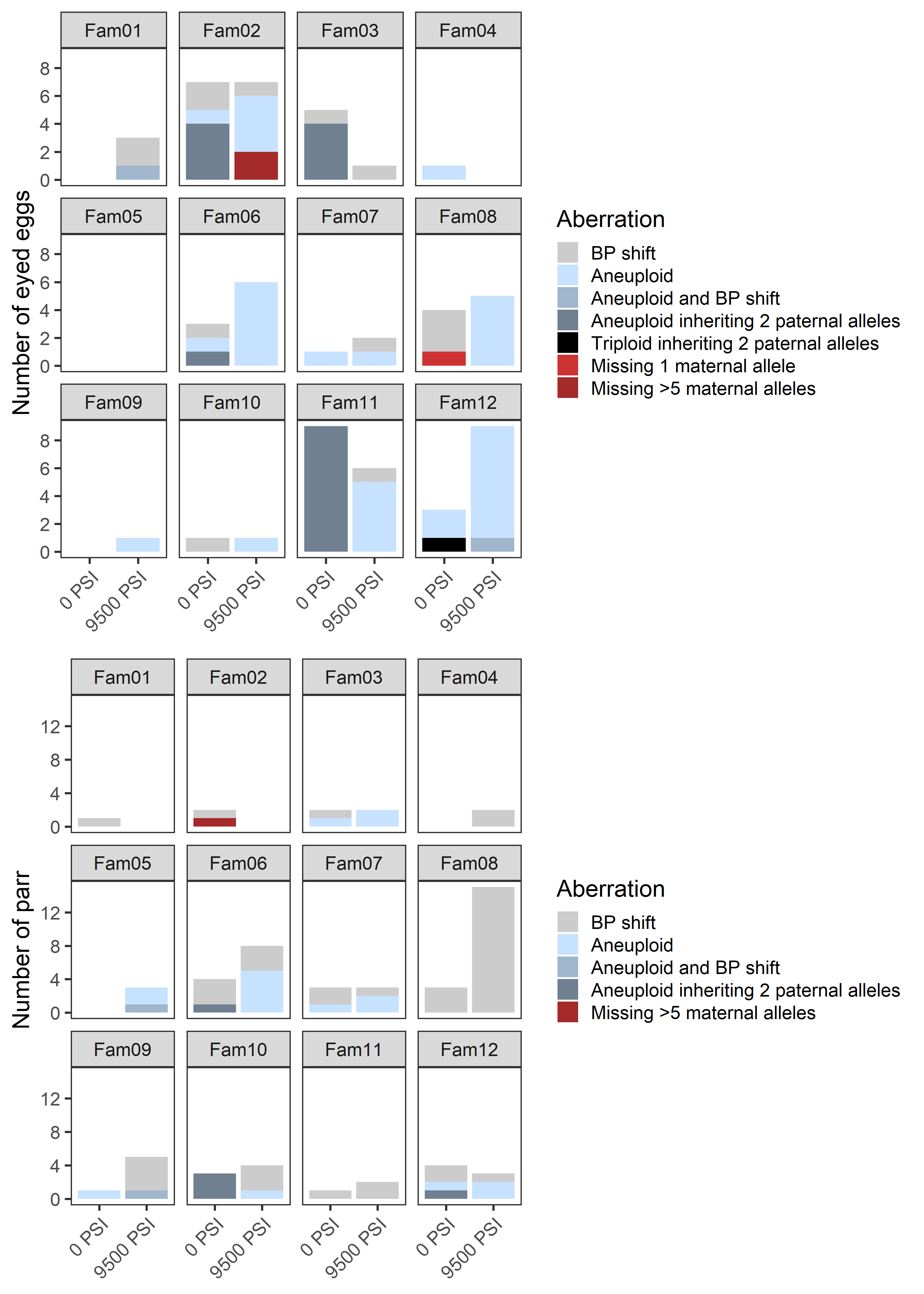


*Supplementary Figure 5: Incidence of inheritance aberrations among eyed eggs (top, N=472) and surviving parr (bottom, N=2,179) from the diploid (0 PSI) and triploid (9500 PSI) pressure treatments (Experiment 2), grouped by family.*

*
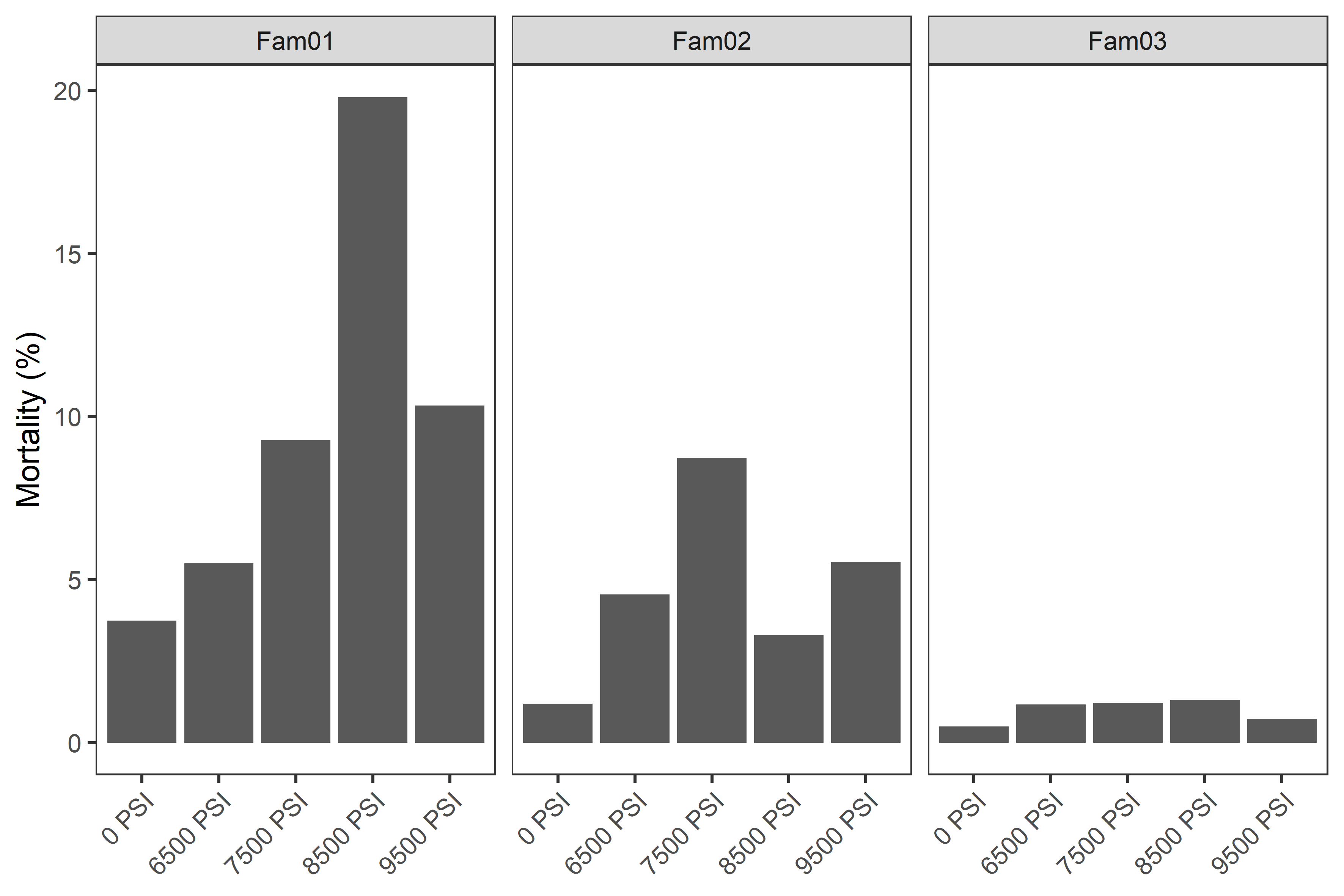
*

*Supplementary Figure 6: Mortality rates from fertilization until the eyed egg stage in Atlantic salmon from three half-sibling families subjected to five hydrostatic pressure treatments (Experiment 1).*

*
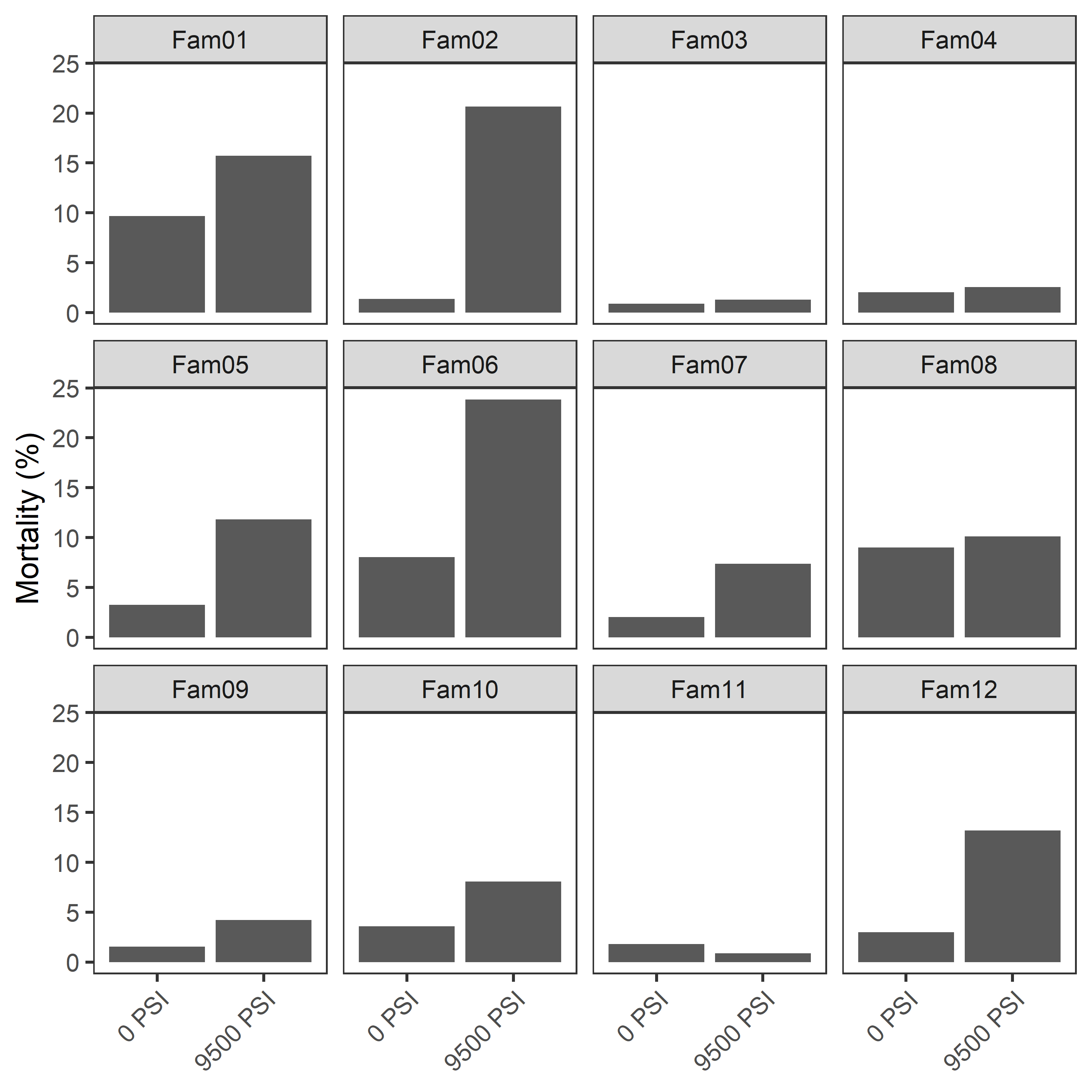
*

*Supplementary Figure 7: Mortality rates from fertilization until the eyed egg stage in Atlantic salmon from 12 families subjected to two hydrostatic pressure treatments (Experiment 2).*
